# Identification of bicalutamide resistance-related genes and prognosis prediction in patients with prostate cancer

**DOI:** 10.3389/fendo.2023.1125299

**Published:** 2023-04-18

**Authors:** Yuezheng Li, Haoyu Wang, Yang Pan, Shangren Wang, Zhexin Zhang, Hang Zhou, Mingming Xu, Xiaoqiang Liu

**Affiliations:** Department of Urology, Tianjin Medical University General Hospital, Tianjin, China

**Keywords:** prostate cancer, androgen deprivation therapy, bicalutamide, immune infiltration, prognostic model

## Abstract

**Background:**

Prostate cancer (PCa) is the second most common type of cancer and the fifth leading cause of cancer-related death in men. Androgen deprivation therapy (ADT) has become the first-line therapy for inhibiting PCa progression; however, nearly all patients receiving ADT eventually progress to castrate-resistant prostate cancer. Therefore, this study aimed to identify hub genes related to bicalutamide resistance in PCa and provide new insights into endocrine therapy resistance.

**Methods:**

The data were obtained from public databases. Weighted correlation network analysis was used to identify the gene modules related to bicalutamide resistance, and the relationship between the samples and disease-free survival was analyzed. Gene Ontology and Kyoto Encyclopedia of Genes and Genomes analyses were performed, and hub genes were identified. The LASSO algorithm was used to develop a bicalutamide resistance prognostic model in patients with PCa, which was then verified. Finally, we analyzed the tumor mutational heterogeneity and immune microenvironment in both groups.

**Results:**

Two drug resistance gene modules were identified. Gene Ontology and Kyoto Encyclopedia of Genes and Genomes analyses revealed that both modules are involved in RNA splicing. The protein–protein interaction network identified 10 hub genes in the brown module *LUC7L3*, *SNRNP70*, *PRPF3*, *LUC7L*, *CLASRP*, *CLK1*, *CLK2*, *U2AF1L4*, *NXF1*, and *THOC1*) and 13 in the yellow module (*PNN*, *PPWD1*, *SRRM2*, *DHX35*, *DMTF1*, *SALL4*, *MTA1*, *HDAC7*, *PHC1*, *ACIN1*, *HNRNPH1*, *DDX17*, and *HDAC6*). The prognostic model composed of *RNF207*, *REC8*, *DFNB59*, *HOXA2*, *EPOR*, *PILRB*, *LSMEM1*, *TCIRG1*, *ABTB1*, *ZNF276*, *ZNF540*, and *DPY19L2* could effectively predict patient prognosis. Genomic analysis revealed that the high- and low-risk groups had different mutation maps. Immune infiltration analysis showed a statistically significant difference in immune infiltration between the high- and low-risk groups, and that the high-risk group may benefit from immunotherapy.

**Conclusion:**

In this study, bicalutamide resistance genes and hub genes were identified in PCa, a risk model for predicting the prognosis of patients with PCa was constructed, and the tumor mutation heterogeneity and immune infiltration in high- and low-risk groups were analyzed. These findings offer new insights into ADT resistance targets and prognostic prediction in patients with PCa.

## 1 Introduction

Prostate cancer (PCa) is the second most common cancer and the fifth leading cause of cancer-related death in men. In 2022, approximately 1,414,259 new cases of PCa were diagnosed worldwide, and 375,304 deaths were reported (1). It is expected that the number of new cases of PCa worldwide and that of deaths will increase to approximately 1.7 million and 499,000, respectively, by 2030 (2). The growth and development of the prostate depend on androgens, which play a predominant role in the development of PCa (3). Therefore, androgen deprivation therapy (ADT) is the first-line therapy for inhibiting PCa progression. Despite an 80–90% initial efficacy, virtually all patients receiving ADT eventually develop castrate-resistant prostate cancer (CRPC) (4). The data indicate that the median survival of patients with CRPC is about 14 months (range 9–30). Moreover, about 15–33% of patients with non-metastatic CRPC would develop metastases within 2 years, contributing to the mortality load of PCa (5). Thus, it is crucial to understand the mechanism of ADT resistance and identify related therapeutic targets to help improve the prognosis of patients with PCa.

Among ADTs, bicalutamide belongs to the first generation of non-steroidal antiandrogen drugs, which can effectively block androgen receptor (AR) activity and tumor invasion in androgen-responsive PCa and has been widely used in clinical practice (6). Over time, drug resistance has emerged in patients with PCa. Recent studies have found that AR mutations, protocadherin B9, and the microtubule-associated protein tau contribute to bicalutamide resistance (7–9). However, the underlying mechanisms remain unclear. Therefore, identifying biomarkers of bicalutamide resistance and potential therapeutic targets may greatly contribute to choosing treatment options.

With the advent of high-throughput sequencing and bioinformatics, researchers can classify and analyze a large number of samples, explore tumor characteristics and heterogeneity, and find new personalized markers. Based on these methods, hub genes associated with PCa progression and recurrence and CRPC have been identified (10, 11). However, only a few studies have used bioinformatics to explore the key genes and mechanisms underlying ADT resistance in PCa.

In the present study, our purpose is identifying hub genes related to bicalutamide resistance in PCa. These genes may be related to endocrine therapy resistance in patients with PCa and may be potential targets for reversing such resistance. Later we constructed a risk model to predict the prognosis of patients with PCa based on samples and analyzed tumor mutational heterogeneity and immune infiltration in high- and low-risk patient groups. Altogether, our findings provide new insights into ADT resistance and prognostic prediction in patients with PCa, which will help establish personalized treatment regimens and drug choice.

## 2 Materials and methods

### 2.1 Data acquisition

Transcriptome and clinical data from The Cancer Genome Atlas - Prostate Adenocarcinoma (TCGA-PRAD) dataset were downloaded from the Xena database (https://xena.ucsc.edu/). After excluding samples without disease-free survival (DFS), Gleason score, and T stage, 483 samples were included in this study. RNA-seq data were converted to transcripts per million to remove the effect of sequencing depth. TCGA-PRAD single nucleotide mutation data were downloaded from the Genomic Data Commons - The Cancer Genome Atlas website (https://portal.gdc.cancer.gov/). A total of 1832 differentially expressed genes in PCa were obtained from the GEPIA2 database (http://gepia.cancer-pku.cn/detail.php?clicktag=degenes).

### 2.2 Weighted correlation network analysis and predictive analysis of drugs

The pRRophetic package (12) was used to analyze the transcriptome data of the 483 samples to predict the bicalutamide resistance of each sample. Subsequently, we performed WGCNA (13) to further identify the gene modules related to drug resistance. We set a soft threshold of 10, and each gene module included at least 30 genes. Finally, 49 samples were discarded owing to outliers, and 6 gene modules were obtained. Univariate Cox regression analysis was used to predict the relationship with DFS in the sample modules.

### 2.3 Gene ontology and Kyoto encyclopedia of genes and genomes pathway enrichment analyses

GO and KEGG analyses of the brown (218) and yellow modules (163) were annotated using the Metascape (14) website (http://metascape.org/gp/index.html#/main/step1). Concomitantly, the key hub genes in these modules were identified *via* the protein–protein interaction network (PPI). These key hub genes were identified with the MCODE plugin of Cytoscape, and statistical significance was set at p < 0.05.

### 2.4 Prognostic model establishment

To further determine the prognostic model for drug resistance genes, univariate Cox regression analysis was used for all genes in the brown and yellow modules, and 89 prognostic genes were identified. We included these genes in the prognostic model, which consisted of 12 genes and was constructed using the LASSO algorithm. The risk score was calculated as (risk coefficient × gene expression level). Subsequently, the samples were divided into a training set and a validation set at a 1:1 ratio, and the low- and high-risk groups were divided by the median risk score. The Kaplan–Meier (KM) curve was used to describe the DFS of the low- and high-risk groups, and statistical significance was set at p < 0.05. Receiver-operating characteristic (ROC) curves were used to demonstrate the predictive efficacy of the training and validation sets for 1, 3, and 5 years.

### 2.5 Mutational landscape diagram

The single-nucleotide mutation data of TCGA-PRAD were processed using the maftools package (15). The 10 most mutated genes were determined for the high- and low-risk groups.

### 2.6 Immunoassay

Twenty-eight tumor immune cell markers were obtained from published articles and seventeen immune pathways were obtained from the IMMPORT website (https://www.immport.org/home). The ssGSEA algorithm (16) was used to calculate the enrichment scores of the 28 immune cells and 17 immune pathways in the sample. The Wilcoxon test was used to identify the difference between the immune cell fractions and pathway scores of the high- and low-risk groups. Furthermore, the expression of 39 immune checkpoint molecules was also examined for differences between these two groups using the Wilcoxon test, and statistical significance was set at p < 0.05.

### 2.7 Statistical analysis

All statistical analyses were performed using R version 4.1.1. Specific statistical methods are referred to in the above Methods subsections.

## 3 Results

### 3.1 Identification of gene modules associated with bicalutamide resistance

**Figure 1:**
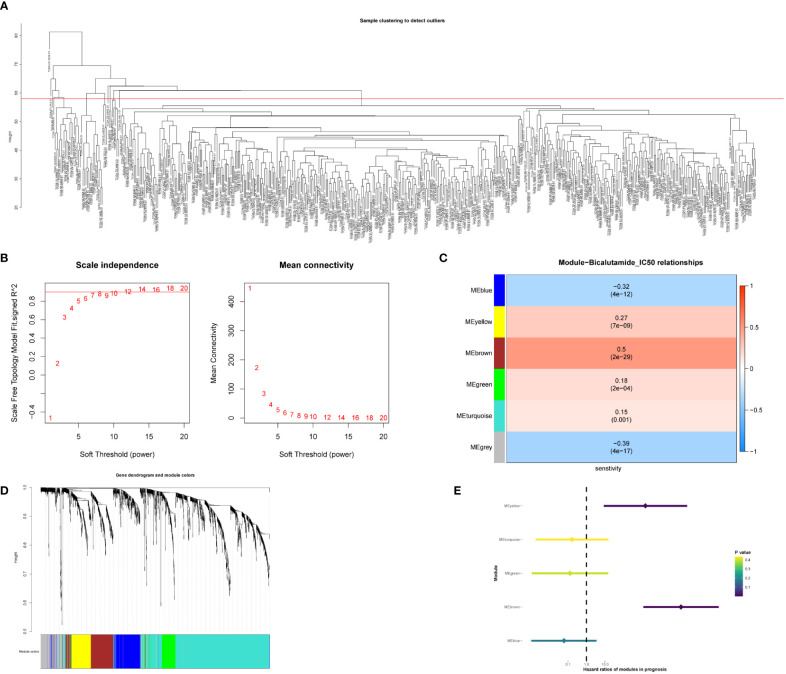
Identification of gene modules associated with bicalutamide resistance. **(A)** Sample clustering. **(B)** Scale-free fit index for various soft-thresholding powers. Mean connectivity for various soft-thresholding powers. **(C)** Dendrogram of all differentially expressed genes clustered based on dissimilarity. **(D)** Correlation between the 6 gene modules with the bicalutamide IC50 values. **(E)** Forest plot of univariate survival analysis for the 6 modules.

Identification of bicalutamide resistance-related genes can help urologists personalize drug choice for patients with PCa. To identify key genes, we calculated the half-maximal inhibitory concentration (IC50) of bicalutamide for each sample in the TCGA-PRAD dataset. WGCNA was constructed based on 1832 differentially expressed genes in PCa. A total of 434 samples were included in subsequent analyses, while 49 were excluded (**Figure 1A**). In the scale-free network, the soft threshold was set to 10 (**Figure 1B**). The gene matrix was transformed into an adjacency matrix and an adjacency topology matrix. At least 30 genes were identified in each module. The characteristic genes of each module were calculated and the close modules were integrated into a new module. WGCNA identified six gene modules, as shown in **Figure 1C**. Subsequently, we calculated the correlation between each module, each sample, and the IC50 values for bicalutamide (**Figure 1D** and **Supplementary Table 1**). The brown and yellow modules displayed strong positive correlations with the IC50 values for bicalutamide (r = 0.5, p = 2e−29; r = 0.27, p = 7e−09). The brown and yellow genes are shown in **Supplementary Tables 2**, **3**, respectively. Subsequently, we used univariate Cox regression to analyze the correlation between the gene expression of each module and DFS. The brown and yellow gene modules were highly correlated with the prognosis of patients with PCa (HR > 1, **Figure 1E**). In summary, we identified brown and yellow gene modules that were closely associated with the IC50 values of bicalutamide and DFS, suggesting that these two gene modules may be associated with resistance to endocrine therapy.

### 3.2 GO and KEGG analyses

**Figure 2:**
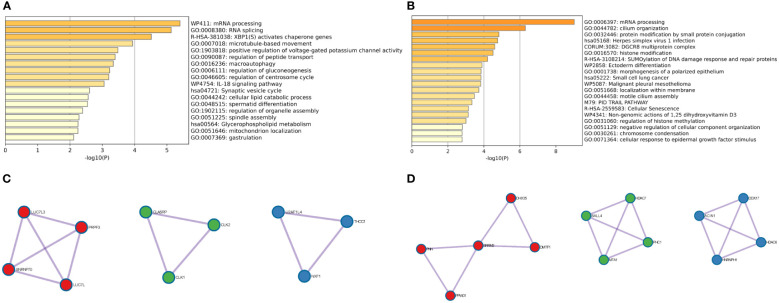
GO and KEGG enrichment analyses of bicalutamide-resistance genes and hub gene identification. **(A)** GO and KEGG analyses of the brown module. **(B)** Hub genes of the brown module. **(C)** GO and KEGG analyses of the yellow module. **(D)** Hub genes of the yellow module.

To further identify the biological processes in which genes in the brown and yellow modules are involved, GO and KEGG enrichment analyses were carried out. As shown in **Figure 2A**, the genes in the brown module were mainly involved in mRNA processing, RNA splicing, XBP1(S) activation of chaperone genes, and microtubule-based movement. PPI analysis showed that 10 genes (*LUC7L3, SNRNP70, PRPF3, LUC7L, CLASRP, CLK1, CLK2, U2AF1L4, NXF1*, and *THOC1*) are hub genes of the brown module (**Figure 2B**). Conversely, the genes in the yellow module were mainly related to mRNA processing, cilium organization, protein modification by small protein conjugation, and herpes simplex virus 1 (HSV-1) infection (**Figure 2C**). The 13 hub genes in the yellow module include were *PNN, PPWD1, SRRM2, DHX35, DMTF1, SALL4, MTA1, HDAC7, PHC1, ACIN1, HNRNPH1, DDX17*, and *HDAC6* (**Figure 2D**). Taken together, both brown and yellow module genes are involved in RNA splicing, suggesting that RNA splicing may be an important factor in endocrine resistance. These hub genes may be potential targets for reversing resistance to endocrine therapy.

### 3.3 Establishing a prognostic risk model

**Figure 3:**
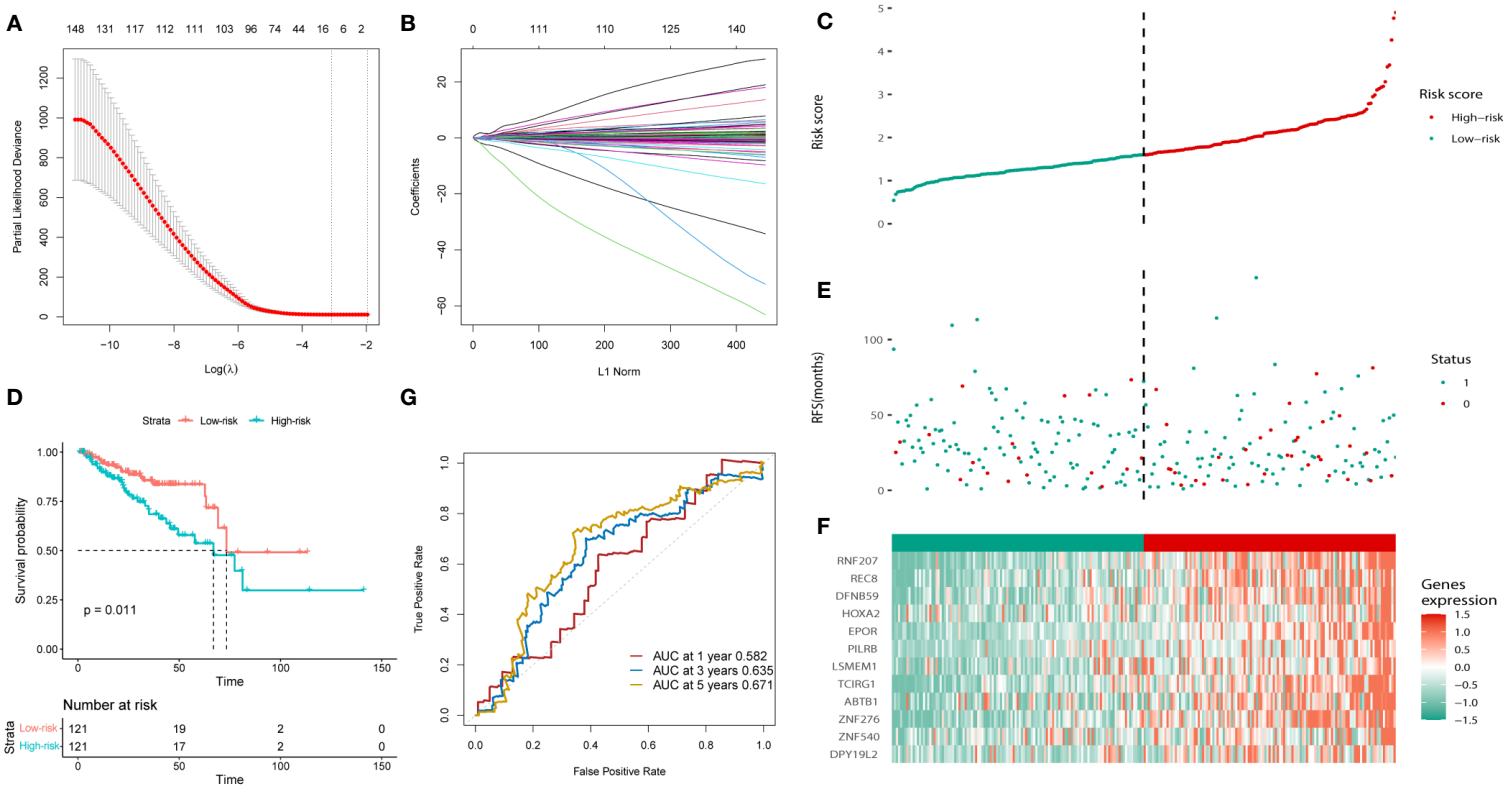
Construction of the bicalutamide-resistance gene-based risk prognostic model in the training cohort. **(A, B)** Construction of the LASSO regression model based on the 12 predictive genes. **(C)** Distribution of the risk scores. **(D)** The KM analysis of PFS in the high- and low-risk groups. **(E)** Survival status. **(F)** Expression of the 12 predictive genes. **(G)** ROC analysis to evaluate the predictive efficiency.

Univariate Cox regression analysis was used to calculate the prognostic risk of the brown and yellow module genes (**Supplementary Table 4**), which yielded 204 genes with survival values. A prognostic model was constructed using LASSO regression analysis (**Figures 3A, B**). The obtained prognostic model comprised *RNF207, REC8, DFNB59, HOXA2, EPOR, PILRB, LSMEM1, TCIRG1, ABTB1, ZNF276, ZNF540*, and *DPY19L*. All the included samples were randomly divided into a training cohort and a test cohort (242:241), and the training cohort was divided into low- and high-risk groups according to the median risk score (121:121) (**Figure 3C**). The KM curve in the training cohort showed that high-risk patients had a worse prognosis than low-risk patients (p = 0.011, **Figure 3D**). More patients in the high-risk group suffered recurrence or death, and a shorter survival period (**Figure 3E**). Heat map analysis revealed elevated expression levels of the 12 risk genes in high-risk patients (**Figure 3F**). Subsequently, ROC curves were used to evaluate the prognostic efficacy of the 12-gene in patients with PCa. As shown in **Figure 3G**, the area under the curve (AUC) scores of the 12 genes prognostic model in the training cohort for 1-, 3-, and 5-year survival prediction were 0.582, 0.635, and 0.671, respectively.

### 3.4 Validation of the prognostic model

**Figure 4:**
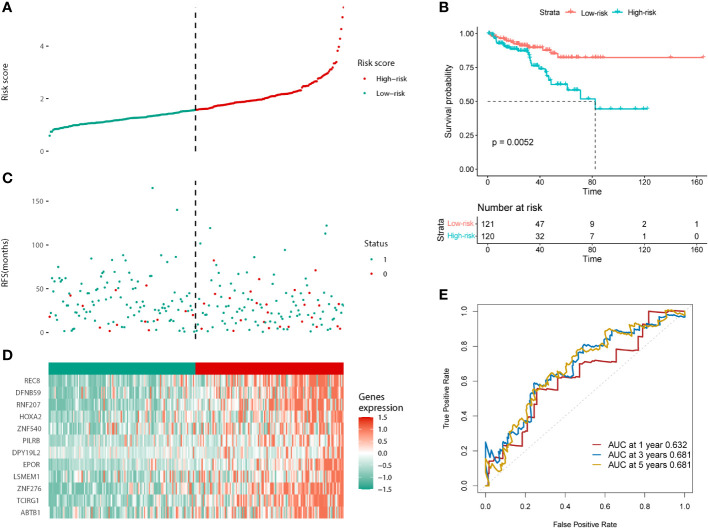
Validation of the prognostic model in the test cohort. **(A)** Distribution of the risk scores. **(B)** The KM analysis of PFS in the high and low-risk groups. **(C)** Survival status. **(D)** Expression of the 12 predictive genes. **(E)** ROC analysis to evaluate the predictive efficiency.

The same results were validated in the test cohort, which was divided into low and high-risk groups according to the median risk score (121:120; **Figure 4A**). The KM curves for the test cohort are shown in **Figure 4B** (p = 0.0052). The high-risk group tended to have a worse prognosis (recurrence or death) and higher risk of gene expression (**Figures 4C, D**). The AUC scores of the prognostic model for predicting 1-, 3-, and 5-year survival in the test cohort were 0.632, 0.681, and 0.681, respectively (**Figure 4E**). In conclusion, the prognostic model we constructed can predict the prognosis of patients with PCa.

### 3.5 Mutational landscape diagram

**Figure 5:**
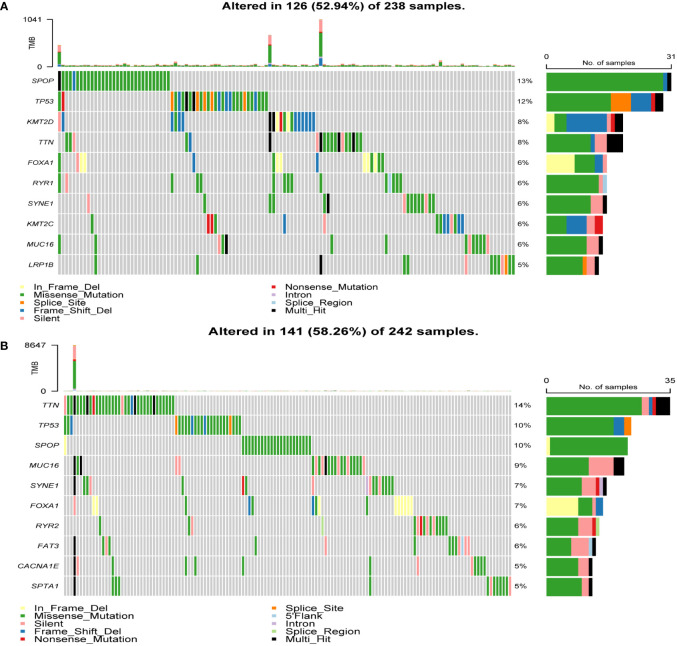
Heterogeneity of tumor mutations between the high and low-risk groups. **(A)** Mutational landscape diagram in the high-risk group. **(B)** Mutational landscape diagram in the low-risk group.

To determine the heterogeneity between the high- and low-risk groups in patients with PCa, we studied the mutation landscape diagram of the two groups. By displaying the 10 most mutated genes in the identified PRAD database, we observed a significantly different landscape between the high- and low-risk groups (**Figures 5A, B**). The frequencies of *SPOP* and *TP53* mutations were significantly higher in the high-risk group than in the low-risk group (13% vs. 10% and 12% vs. 10%, respectively), while the *TTN* mutation rate was significantly lower (8% vs. 14%). The high-risk group also presented mutations in *KMT2D, FOXA1*, and *RYR1* (8%, 6%, and 6%, respectively). Conversely the low-risk group presented *MUC16, SYNE1*, and *FOXA1* mutations (9%, 7%, and 7%, respectively). In summary, genomic heterogeneity was identified between the low- and high-risk populations.

### 3.6 Immune infiltration analysis

**Figure 6:**
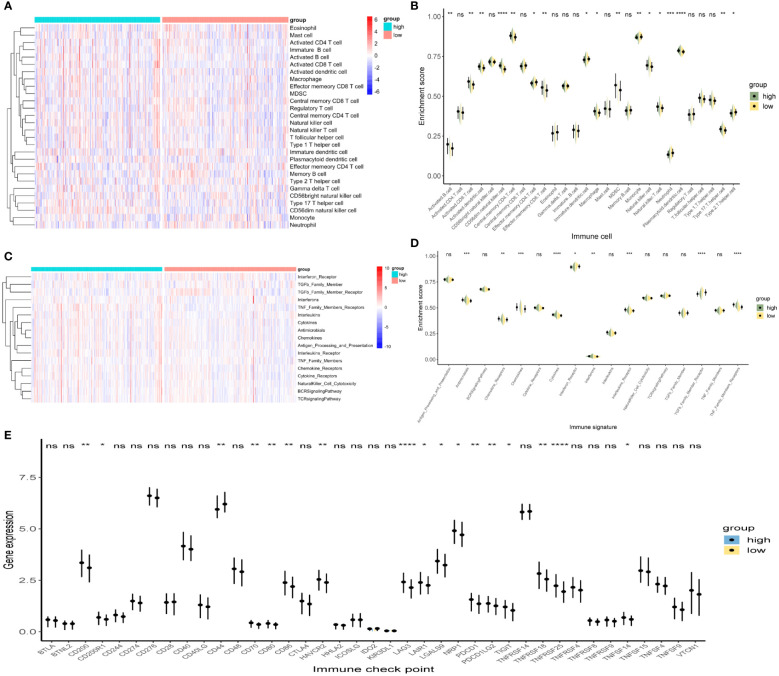
Immune infiltration analysis between the high- and low-risk groups. **(A, B)** Heat map and violin plot of 28 immune cells. **(C, D)** Heat map and violin plot resulting from the enrichment analysis of 17 immune-related signaling pathways. **(E)** Expression levels of immune checkpoints. *p < 0.05, **p < 0.01, ***p < 0.001, ****p < 0.0001, whereas ‘ns’ is non-significant.

In tumors, the immune microenvironment is closely related to endocrine resistance (17). Therefore, we assessed the presence of 28 immune cell types in TCGA-PRAD samples (**Figure 6A**). Violin plots showed that the high-risk group had a higher immune infiltration (**Figure 6B**). Activated B cells, activated CD8 T cells, CD56dim natural killer (NK) cells, central memory CD4 T cells, and plasmacytoid dendritic cells were significantly increased in the high-risk group. We also assessed the presence of 17 immune-related signaling pathways (**Figure 6C**). The violin plot shows that antimicrobials, chemokines, cytokines, and TNF family members receptors are increased in the high-risk group (**Figure 6D**). Finally, we analyzed the expression levels of immune checkpoints in the two groups (**Figure 6E**). CD200, CD200R1, CD86, LAG3, LAIR1, LGALS9, NRP1, TIGIT, TNFRSF18, TNFRSF25, and TNFSF14 were significantly upregulated in the high-risk group. In summary, the high- and low-risk groups showed different immune infiltration, and the high expression of immune checkpoint molecules in the high-risk group suggests that the high-risk group may benefit from immune checkpoint blockade.

## 4 Discussion

PCa is a solid malignant tumor in males with high morbidity, mortality, and heterogeneity (18). ADT is the treatment of choice for PCa at virtually all stages (19). Nonetheless, almost all patients receiving ADT eventually develop CRPC (4), thereby hindering the therapeutic efficacy of the initial ADT plan. Consequently, it is crucial to identify genes related to ADT resistance in PCa and explore the mechanisms of this resistance. To date, only a few studies have been conducted in this direction.

In this study, through a comprehensive analysis of transcriptome data and bicalutamide IC50 values of samples from the TCGA-PRAD dataset, we identified two gene modules, brown and yellow, that are associated with bicalutamide treatment resistance. To explore how these genes are involved in bicalutamide resistance in patients with PCa, GO and KEGG enrichment analyses were performed. The results showed that these genes are mainly involved in mRNA processing and RNA splicing, and are also involved in the XBP1(S) activates chaperone genes, microtubule-based movement, positive regulation of voltage-gated potassium channel activity, macroautophagy, protein modification by small protein conjugation, and HSV-1 infection. Previous studies have found that many aberrant mRNA splice variants are upregulated in PCa, which further aggravates the disease by promoting proliferation, metastasis, tumor growth, anti-apoptosis, and drug resistance (20). Selective cleavage of AR is an important mechanism of drug resistance in PCa. Among the 20 different AR splice variants identified, Arv7 is the most common (21). ARv7 mRNA levels in patients with PCa have been shown to help predict responsiveness to ADTs, such as enzalutamide and abiraterone (22). C-MYC signaling is highly activated in the progression of PCa, which needs XBP1(S). Expression of XBP1(S) is strongly correlated with PCa prognosis (23). Zucchini et al. found that nine functional groups associated with high liver/bone/kidney alkaline phosphatase activity, including microtubule movement, are strongly associated with tumor aggressiveness (24). Voltage-gated potassium channels can regulate cancer cell proliferation, and their inhibition *via* piperine can have a therapeutic effect in PCa (25). Nguyen et al. found that autophagy is an important mechanism of CRPC resistance to AR inhibitors (such as bicalutamide and enzalutamide), and blocking autophagy significantly reduced the survival of PCa cells *in vitro* and *in vivo*, suggesting the great potential of autophagy inhibitors in the treatment of patients with CRPC (26). Tokarz et al. developed inhibitors of small ubiquitin related modified protein (SUMO) specific proteases (SENPs). *In vitro* and *in vivo* experiments have shown that SENPs are a suitable target for anti-tumor therapy (27). HSV-1 is also involved in tumorigenesis (28). Therefore, we speculate that the brown and yellow module genes play an important role in drug resistance and PCa progression through these pathways.

We performed PPI analysis of the two resistance-related gene modules separately, and identified 23 hub genes (*LUC7L3, SNRNP70, PRPF3, LUC7L, CLASRP, CLK1, CLK2, U2AF1L4, NXF1*, *THOC1, PNN, PPWD1, SRRM2, DHX35, DMTF1, SALL4, MTA1, HDAC7, PHC1, ACIN1, HNRNPH1, DDX17*, and *HDAC6*). *HNRNPH1* knockdown has been shown to reduce the expression of AR and its splice variant AR-V7 (or AR3). Small interfering RNA silencing of *HNRNPH1* sensitizes PCa cells to bicalutamide and inhibits prostate tumorigenesis *in vivo* (29). HDAC6 deacetylates various cytoplasmic proteins and participates in protein degradation, protein transport, cell migration, and metastasis. Zhou et al. studied a novel AR/HDAC6 dual inhibitor, which showed a more potent anti-proliferative effect on PCa cells than an AR antagonist (MDV3100) (30). High expression levels of *THOC1* (31), *PNN* (32), *MTA1* (33), *DDX17* (34), and *CLK1* (35) have been shown to promote PCa progression. Niklaus et al. found that *DMTF1* expression is related to increased cisplatin resistance in breast cancer (36). Therefore, we believe that these 23 hub genes may be important targets for reversing ADT resistance in patients with PCa, and that part of these can also improve the sensitivity of patients to chemotherapy.

Patients with PCa with castration resistance present a shorter survival time and higher risk of progression (4). For survival prediction, we analyzed the prognostic risk of the brown and yellow gene modules, and finally established a prognostic model consisting of *RNF207, REC8, DFNB59, HOXA2, EPOR, PILRB, LSMEM1, TCIRG1, ABTB1, ZNF276, ZNF540*, and *DPY19L2*. According to the median risk score, the patients were divided into high- and low-risk groups. The results showed that the high-risk group had a higher number of recurrences or deaths and shorter survival than the low-risk group. A high expression of these 12 genes is related to a worse prognosis. *RNF207* was found to predict lymph node involvement in patients with obesity and endometrial cancer (37). By targeting the PKA pathway, *REC8* can promote tumor migration, invasion, and angiogenesis in hepatocellular carcinoma (38). *PILRB* (39) and *TCIRG1* (40) are associated with clear cell renal cell carcinoma prognosis. Huang et al. found that targeted downregulation of *ABTB1* expression *via* miR-4319 can inhibit colorectal cancer progression (41). *ZNF276* can promote the malignant phenotype of breast cancer by activating the CYP1B1-mediated Wnt/β-catenin pathway (42). Subsequently, we used the test cohort to validate the model, and the results were highly consistent with the training cohort, implying that our prognostic model can predict the prognosis of patients with PCa.

Tumor mutation burden is closely associated with tumor heterogeneity (43). To determine the heterogeneity of the high- and low-risk groups in patients with PCa, we studied the mutation landscape diagram in both groups; the most frequently mutated genes were *SPOP* and *TTN* in the high- and low-risk groups, respectively, which may indicate that patients with a high *SPOP* mutation rate have a worse prognosis, whereas those with a high *TTN* mutation rate have a relatively better prognosis. *TP53* was the second most frequent mutation in both groups. *SPOP* mutations can promote PCa progression by promoting autophagy and Nrf2 activation (44). However, *SPOP* mutation can increase the sensitivity of PCa cells to ADT (45). Studies have shown that patients with metastatic CRPC and *SPOP* mutations and/or CHD1 deletions are more sensitive to abiraterone treatment (46). Notably, *SPOP* mutations lead to PCa resistance to cellular stress induced by chemotherapeutic agents such as docetaxel (47). *TP53* is the most prominent gene in pan-cancer studies, and its somatic alterations are independently associated with the rapid emergence of drug resistance in patients with metastatic CRPC (48). Mutations or deletions in *TP53* and *RB1* can transform PCa AR-dependent luminal epithelial cells into AR-independent basal-like cells, which are resistant to ADT (49). These genes may be potential targets for the prognosis of PCa, and the different gene mutation frequencies between the high- and low-risk groups may provide new therapeutic strategies for PCa endocrine resistance.

Further research, has shown that the tumor immune microenvironment is closely associated with endocrine therapy resistance (17). Therefore, we assessed the abundance of immune cells and immune-related signaling pathways in the high- and low-risk groups as well as the level of immune checkpoint expression. In the high-risk group, we found that activated B cells, activated CD8 T cells, macrophages, and NK cells were highly expressed, and antimicrobials, chemokines, cytokines, and TNF family members receptors were up-regulated. Loss of NK cell activity has a significant correlation with PCa progression and the lethal phenotype of metastatic CRPC (50). NK cells inhibit enzalutamide resistance and cell invasion in CRPC by targeting ARv7 (51). Prostate tumor-associated macrophages can promote the growth of PCa, and secreted Gas6 can further enhance the activation of RON and AXL receptors in PCa cells, thereby driving CRPC. Targeting RON and macrophages promotes CRPC sensitivity to ADT (52). Targeting the CSF1 receptor can also reverse macrophage-mediated resistance to androgen blockade in PCa (53). IL-23 produced by myeloid-derived suppressor cells (MDSCs) can activate the AR pathway in PCa cells, and promote cell survival and proliferation under androgen-deprived conditions. Treatment that blocks IL-23 antagonizes MDSC-mediated castration resistance in PCa (54). CXCR7 activates MAPK/ERK signaling, which contributes to enzalutamide resistance in PCa (55). The combination of enzalutamide and a CXCR7 inhibitor can reduce pro-angiogenic signaling and macro-angiogenesis in PCa, and its therapeutic effect is significantly better than that of enzalutamide monotherapy (56). IL-6 can induce the castration-resistant growth of androgen-dependent human PCa cells and increase the bicalutamide resistance of PCa cells through TIF2 (57). These results also showed that the high- and low-risk groups had a different immune status.

After immune checkpoint expression analysis, we found that CD200, CD200R1, CD70, CD80, CD86, HAVCR2, LAG3, LAIR1, LGALS9, NRP1, PDCD1, PDCD1LG2, TIGIT, TNFRSF18, TNFRSF25, and TNFSF14 were highly expressed in the high-risk group. Based on the inflammation and immune imbalance in various tumor microenvironments, the CD200–CD200R pathway is differentially regulated (58). In liver metastases from primary pancreatic ductal adenocarcinoma, CD200 and BTLA pathways can drive macrophage-mediated adaptive immune tolerance. Targeting CD200/BTLA can enhance the immunogenicity of macrophages and T cells and enhance the immunotherapeutic effect on liver metastases (59). Allogeneic CAR-T cells targeting CD70 have shown efficacy in the treatment of renal cell carcinoma and have entered phase I clinical trials (60). Cis-PD-L1 interacts with CD80 to obtain an optimal T-cell response to destroy the tumor (61). LAG3 can be used as a target for cancer immunotherapy, targeting LAG3/GAL-3 to overcome immunosuppression and enhances the antitumor immune response in multiple myeloma (62). Blockade of Sema3A/Nrp1 signaling prevents macrophages from entering hypoxic tumor regions, inhibits angiogenesis and restores anti-tumor immunity (63). Inhibition of TIGIT enhances the functional activity of NK cells against CRPC cells (64). These studies suggest that patients in high-risk groups may benefit from immunotherapy targeting these checkpoints.

Notably, this study has some strengths. Firstly, 23 hub genes associated with bicalutamide resistance were identified. These genes may be potential targets for reversing bicalutamide resistance or even other endocrine therapies resistance in PCa. Then, we constructed a prognostic model consisting of 12 genes, showing a high predictive value. Besides, we found differential tumor mutation burden and immune status in high- and low-risk groups. Therefore, our study has greater clinical implications for the prognostic assessment and selection of treatment options for patients with PCa. Yet, our study presents certain limitations. First of all, it’s a retrospective study with relatively small sample sizes in the training and test cohorts, so further studies with larger cohorts are necessary to confirm our results. Moreover, *in vivo* and *in vitro* experiments are required, and the function of drug resistance genes needs to be further explored.

## 5 Conclusion

In summary, using public databases, we identified genes and hub genes associated with bicalutamide resistance in PCa. These genes may be related to endocrine therapy resistance in PCa, and may be potential targets for reversing endocrine therapy resistance. Concomitantly, we constructed an effective risk model to predict the prognosis of patients with PCa and analyzed tumor mutation heterogeneity and immune infiltration in high- and low-risk groups. In conclusion, this study provides new insights for the exploration of ADT resistance targets and prognosis prediction in patients with PCa, which will help establish personalized treatment options and drug choice.

## Data availability statement

Publicly available datasets were analyzed in this study. This data can be found here: https://xena.ucsc.edu/, the Xena database https://portal.gdc.cancer.gov/, the Genomic Data Commons - The Cancer Genome Atlas http://gepia.cancer-pku.cn/detail.php?clicktag=degenes, the GEPIA2 database.

## Author contributions

YL and XL designed the study. YL, YP, and ZZ performed the bioinformatics analysis. HW, HZ, and MX wrote the manuscript. SW and XL supervised the study. All authors have read and approved the final manuscript. All authors contributed to the article and approved the submitted version.

## Supplementary material

Supplementary Table 1 The correlation analysis between each sample and IC50 values for bicalutamide.

Supplementary Table 2 List of genes in the brown module.

Supplementary Table 3 List of genes in the yellow module.

Supplementary Table 4 Univariate Cox regression analysis of prognostic risk for genes in the brown and yellow modules.

## References

[1] Sung H Ferlay J Siegel RL Laversanne M Soerjomataram I Jemal A. Global cancer statistics 2020: GLOBOCAN estimates of incidence and mortality worldwide for 36 cancers in 185 countries. CA: Cancer J Clin (2021) 71(3):209–49. doi: 10.3322/caac.21660 33538338

[2] Taitt HE. Global trends and prostate cancer: a review of incidence, detection, and mortality as influenced by race, ethnicity, and geographic location. Am J men's Health (2018) 12(6):1807–23. doi: 10.1177/1557988318798279 PMC6199451 30203706

[3] Hsing AW Chu LW Stanczyk FZ. Androgen and prostate cancer: is the hypothesis dead? Cancer epidemiology Biomarkers Prev (2008) 17(10):2525–30. doi: 10.1158/1055-9965.EPI-08-0448 18842992

[4] Wang Y Chen J Wu Z Ding W Gao S Gao Y. Mechanisms of enzalutamide resistance in castration-resistant prostate cancer and therapeutic strategies to overcome it. Br J Pharmacol (2021) 178(2):239–61. doi: 10.1111/bph.15300 33150960

[5] Mansinho A Macedo D Fernandes I Costa L. Castration-resistant prostate cancer: mechanisms, targets and treatment. Adv Exp Med Biol (2018) 1096:117–33. doi: 10.1007/978-3-319-99286-0_7 30324351

[6] Liu C Armstrong CM Lou W Lombard AP Cucchiara V Gu X. Niclosamide and bicalutamide combination treatment overcomes enzalutamide- and bicalutamide-resistant prostate cancer. Mol Cancer Ther (2017) 16(8):1521–30. doi: 10.1158/1535-7163.MCT-16-0912 PMC5544572 28500234

[7] Bohl CE Gao W Miller DD Bell CE Dalton JT. Structural basis for antagonism and resistance of bicalutamide in prostate cancer. Proc Natl Acad Sci United States America. (2005) 102(17):6201–6. doi: 10.1073/pnas.0500381102 PMC1087923 15833816

[8] Sekino Y Oue N Mukai S Shigematsu Y Goto K Sakamoto N. Protocadherin B9 promotes resistance to bicalutamide and is associated with the survival of prostate cancer patients. Prostate. (2019) 79(2):234–42. doi: 10.1002/pros.23728 30324761

[9] Sekino Y Han X Babasaki T Goto K Inoue S Hayashi T. Microtubule-associated protein tau (MAPT) promotes bicalutamide resistance and is associated with survival in prostate cancer. Urologic Oncol (2020) 38(10):795.e1–.e8. doi: 10.1016/j.urolonc.2020.04.032 32430253

[10] Wu YP Ke ZB Lin F Wen YA Chen S Li XD. Identification of key genes and pathways in castrate-resistant prostate cancer by integrated bioinformatics analysis. Pathology Res practice. (2020) 216(10):153109. doi: 10.1016/j.prp.2020.153109 32853947

[11] Li S Hou J Xu W. Screening and identification of key biomarkers in prostate cancer using bioinformatics. Mol Med Rep (2020) 21(1):311–9. doi: 10.3892/mmr.2019.10799 PMC6896273 31746380

[12] Geeleher P Cox N Huang RS. pRRophetic: an r package for prediction of clinical chemotherapeutic response from tumor gene expression levels. PloS One (2014) 9(9):e107468. doi: 10.1371/journal.pone.0107468 25229481 PMC4167990

[13] Langfelder P Horvath S. WGCNA: an r package for weighted correlation network analysis. BMC Bioinf (2008) 9:559. doi: 10.1186/1471-2105-9-559 PMC2631488 19114008

[14] Zhou Y Zhou B Pache L Chang M Khodabakhshi AH Tanaseichuk O. Metascape provides a biologist-oriented resource for the analysis of systems-level datasets. Nat Commun (2019) 10(1):1523. doi: 10.1038/s41467-019-09234-6 30944313 PMC6447622

[15] Mayakonda A Lin DC Assenov Y Plass C Koeffler HP. Maftools: efficient and comprehensive analysis of somatic variants in cancer. Genome Res (2018) 28(11):1747–56. doi: 10.1101/gr.239244.118 PMC6211645 30341162

[16] Hänzelmann S Castelo R Guinney J. GSVA: gene set variation analysis for microarray and RNA-seq data. BMC Bioinf (2013) 14:7. doi: 10.1186/1471-2105-14-7 PMC3618321 23323831

[17] Ruan H Bao L Tao Z Chen K. Flightless I homolog reverses enzalutamide resistance through PD-L1-Mediated immune evasion in prostate cancer. Cancer Immunol Res (2021) 9(7):838–52. doi: 10.1158/2326-6066.CIR-20-0729 34011528

[18] Desai K McManus JM Sharifi N. Hormonal therapy for prostate cancer. Endocr Rev (2021) 42(3):354–73. doi: 10.1210/endrev/bnab002 PMC8152444 33480983

[19] Yu EM Aragon-Ching JB. Advances with androgen deprivation therapy for prostate cancer. Expert Opin Pharmacother. (2022) 23(9):1015–33. doi: 10.1080/14656566.2022.2033210 35108137

[20] Calderon-Aparicio A Wang BD. Prostate cancer: alternatively spliced mRNA transcripts in tumor progression and their uses as therapeutic targets. Int J Biochem Cell Biol (2021) 141:106096. doi: 10.1016/j.biocel.2021.106096 34653618 PMC8639776

[21] Olender J Lee NH. Role of alternative splicing in prostate cancer aggressiveness and drug resistance in African americans. Adv Exp Med Biol (2019) 1164:119–39. doi: 10.1007/978-3-030-22254-3_10 PMC6777849 31576545

[22] Antonarakis ES Lu C Wang H Luber B Nakazawa M Roeser JC. AR-V7 and resistance to enzalutamide and abiraterone in prostate cancer. New Engl J Med (2014) 371(11):1028–38. doi: 10.1056/NEJMoa1315815 PMC4201502 25184630

[23] Sheng X Nenseth HZ Qu S Kuzu OF Frahnow T Simon L. IRE1α-XBP1s pathway promotes prostate cancer by activating c-MYC signaling. Nat Commun (2019) 10(1):323. doi: 10.1038/s41467-018-08152-3 30679434 PMC6345973

[24] Zucchini C Bianchini M Valvassori L Perdichizzi S Benini S Manara MC. Identification of candidate genes involved in the reversal of malignant phenotype of osteosarcoma cells transfected with the liver/bone/kidney alkaline phosphatase gene. Bone. (2004) 34(4):672–9. doi: 10.1016/j.bone.2003.12.008 15050898

[25] George K Thomas NS Malathi R. Piperine blocks voltage gated k(+) current and inhibits proliferation in androgen sensitive and insensitive human prostate cancer cell lines. Arch Biochem biophysics. (2019) 667:36–48. doi: 10.1016/j.abb.2019.04.007 31047869

[26] Nguyen HG Yang JC Kung HJ Shi XB Tilki D Lara PN Jr.. Targeting autophagy overcomes enzalutamide resistance in castration-resistant prostate cancer cells and improves therapeutic response in a xenograft model. Oncogene. (2014) 33(36):4521–30. doi: 10.1038/onc.2014.25 PMC4155805 24662833

[27] Tokarz P Woźniak K. SENP proteases as potential targets for cancer therapy. Cancers (Basel) (2021) 13(9):2059. doi: 10.3390/cancers13092059 33923236 PMC8123143

[28] Golais F Mrázová V. Human alpha and beta herpesviruses and cancer: passengers or foes? Folia microbiologica (2020) 65(3):439–49. doi: 10.1007/s12223-020-00780-x 32072398

[29] Yang Y Jia D Kim H Abd Elmageed ZY Datta A Davis R. Dysregulation of miR-212 promotes castration resistance through hnRNPH1-mediated regulation of AR and AR-V7: implications for racial disparity of prostate cancer. Clin Cancer Res (2016) 22(7):1744–56. doi: 10.1158/1078-0432.CCR-15-1606 PMC7472565 26553749

[30] Zhou M Zheng H Li Y Huang H Min X Dai S. Discovery of a novel AR/HDAC6 dual inhibitor for prostate cancer treatment. Aging. (2021) 13(5):6982–98. doi: 10.18632/aging.202554 PMC7993727 33621955

[31] Chinnam M Wang Y Zhang X Gold DL Khoury T Nikitin AY. The Thoc1 ribonucleoprotein and prostate cancer progression. J Natl Cancer Inst (2014) 106(11):dju306. doi: 10.1093/jnci/dju306 25296641 PMC4271031

[32] Meng XY Zhang HZ Ren YY Wang KJ Chen JF Su R. Pinin promotes tumor progression *via* activating CREB through PI3K/AKT and ERK/MAPK pathway in prostate cancer. Am J Cancer Res (2021) 11(4):1286–303. doi: 10.1093/jnci/dju306 PMC8085840 33948358

[33] Ma J Li C Qian H Zhang Y. MTA1: a vital modulator in prostate cancer. Curr Protein Pept science. (2022) 23(7):456–64. doi: 10.2174/1389203723666220705152713 35792131

[34] Wu XC Yan WG Ji ZG Zheng GY Liu GH. Long noncoding RNA SNHG20 promotes prostate cancer progression *via* upregulating DDX17. Arch Med Sci AMS (2021) 17(6):1752–65. doi: 10.5114/aoms.2019.85653 PMC8641522 34900057

[35] Uzor S Porazinski SR Li L Clark B Ajiro M Iida K. CDC2-like (CLK) protein kinase inhibition as a novel targeted therapeutic strategy in prostate cancer. Sci Rep (2021) 11(1):7963. doi: 10.1038/s41598-021-86908-6 33846420 PMC8041776

[36] Niklaus NJ Humbert M Tschan MP. Cisplatin sensitivity in breast cancer cells is associated with particular DMTF1 splice variant expression. Biochem Biophys Res Commun (2018) 503(4):2800–6. doi: 10.1016/j.bbrc.2018.08.042 30100063

[37] López-Ozuna VM Kogan L Hachim MY Matanes E Hachim IY Mitric C. Identification of predictive biomarkers for lymph node involvement in obese women with endometrial cancer. Front Oncol (2021) 11:695404. doi: 10.3389/fonc.2021.695404 34307159 PMC8292832

[38] Han J Bai Y Wang J Xie XL Li AD Ding Q. REC8 promotes tumor migration, invasion and angiogenesis by targeting the PKA pathway in hepatocellular carcinoma. Clin Exp Med (2021) 21(3):479–92. doi: 10.1007/s10238-021-00698-9 33677646

[39] Chen Y Liang Y Chen Y Ouyang S Liu K Yin W. Identification of prognostic metabolism-related genes in clear cell renal cell carcinoma. J Oncol (2021) 2021:2042114. doi: 10.1155/2021/2042114 34616452 PMC8490028

[40] Xu C Jia B Yang Z Han Z Wang Z Liu W. Integrative analysis identifies TCIRG1 as a potential prognostic and immunotherapy-relevant biomarker associated with malignant cell migration in clear cell renal cell carcinoma. Cancers (Basel) (2022) 14(19):4583. doi: 10.3390/cancers14194583 36230507 PMC9558535

[41] Huang L Zhang Y Li Z Zhao X Xi Z Chen H. MiR-4319 suppresses colorectal cancer progression by targeting ABTB1. United Eur Gastroenterol J (2019) 7(4):517–28. doi: 10.1177/2050640619837440 PMC6488794 31065369

[42] Lei T Zhang W He Y Wei S Song X Zhu Y. ZNF276 promotes the malignant phenotype of breast carcinoma by activating the CYP1B1-mediated wnt/β-catenin pathway. Cell Death disease. (2022) 13(9):781. doi: 10.1038/s41419-022-05223-8 36085146 PMC9463175

[43] Salem ME Bodor JN Puccini A Xiu J Goldberg RM Grothey A. Relationship between MLH1, PMS2, MSH2 and MSH6 gene-specific alterations and tumor mutational burden in 1057 microsatellite instability-high solid tumors. Int J cancer. (2020) 147(10):2948–56. doi: 10.1002/ijc.33115 PMC7530095 32449172

[44] Shi Q Jin X Zhang P Li Q Lv Z Ding Y. SPOP mutations promote p62/SQSTM1-dependent autophagy and Nrf2 activation in prostate cancer. Cell Death differentiation. (2022) 29(6):1228–39. doi: 10.1038/s41418-021-00913-w PMC9177840 34987184

[45] Bernasocchi T El Tekle G Bolis M Mutti A Vallerga A Brandt LP. Dual functions of SPOP and ERG dictate androgen therapy responses in prostate cancer. Nat Commun (2021) 12(1):734. doi: 10.1038/s41467-020-20820-x 33531470 PMC7854732

[46] Boysen G Rodrigues DN Rescigno P Seed G Dolling D Riisnaes R. SPOP-Mutated/CHD1-Deleted lethal prostate cancer and abiraterone sensitivity. Clin Cancer Res (2018) 24(22):5585–93. doi: 10.1158/1078-0432.CCR-18-0937 PMC6830304 30068710

[47] Shi Q Zhu Y Ma J Chang K Ding D Bai Y. Prostate cancer-associated SPOP mutations enhance cancer cell survival and docetaxel resistance by upregulating Caprin1-dependent stress granule assembly. Mol cancer. (2019) 18(1):170. doi: 10.1186/s12943-019-1096-x 31771591 PMC6878651

[48] Annala M Vandekerkhove G Khalaf D Taavitsainen S Beja K Warner EW. Circulating tumor DNA genomics correlate with resistance to abiraterone and enzalutamide in prostate cancer. Cancer discovery. (2018) 8(4):444–57. doi: 10.1158/2159-8290.CD-17-0937 29367197

[49] Mu P Zhang Z Benelli M Karthaus WR Hoover E Chen CC. SOX2 promotes lineage plasticity and antiandrogen resistance in TP53- and RB1-deficient prostate cancer. Sci (New York NY). (2017) 355(6320):84–8. doi: 10.1126/science.aah4307 PMC5247742 28059768

[50] Wu J. Could harnessing natural killer cell activity be a promising therapy for prostate cancer? Crit Rev Immunol (2021) 41(2):101–6. doi: 10.1615/CritRevImmunol.2021037614 PMC8966398 34348004

[51] Lin SJ Chou FJ Li L Lin CY Yeh S Chang C. Natural killer cells suppress enzalutamide resistance and cell invasion in the castration resistant prostate cancer *via* targeting the androgen receptor splicing variant 7 (ARv7). Cancer letters. (2017) 398:62–9. doi: 10.1016/j.canlet.2017.03.035 28373004

[52] Brown NE Jones A Hunt BG Waltz SE. Prostate tumor RON receptor signaling mediates macrophage recruitment to drive androgen deprivation therapy resistance through Gas6-mediated axl and RON signaling. Prostate. (2022) 82(15):1422–37. doi: 10.1002/pros.24416 PMC9492645 35860905

[53] Escamilla J Schokrpur S Liu C Priceman SJ Moughon D Jiang Z. CSF1 receptor targeting in prostate cancer reverses macrophage-mediated resistance to androgen blockade therapy. Cancer Res (2015) 75(6):950–62. doi: 10.1158/0008-5472.CAN-14-0992 PMC4359956 25736687

[54] Calcinotto A Spataro C Zagato E Di Mitri D Gil V Crespo M. IL-23 secreted by myeloid cells drives castration-resistant prostate cancer. Nature. (2018) 559(7714):363–9. doi: 10.1038/s41586-018-0266-0 PMC6461206 29950727

[55] Li S Fong KW Gritsina G Zhang A Zhao JC Kim J. Activation of MAPK signaling by CXCR7 leads to enzalutamide resistance in prostate cancer. Cancer Res (2019) 79(10):2580–92. doi: 10.1158/0008-5472.CAN-18-2812 PMC6522281 30952632

[56] Luo Y Azad AK Karanika S Basourakos SP Zuo X Wang J. Enzalutamide and CXCR7 inhibitor combination treatment suppresses cell growth and angiogenic signaling in castration-resistant prostate cancer models. Int J cancer. (2018) 142(10):2163–74. doi: 10.1002/ijc.31237 PMC5867246 29277895

[57] Feng S Tang Q Sun M Chun JY Evans CP Gao AC. Interleukin-6 increases prostate cancer cells resistance to bicalutamide *via* TIF2. Mol Cancer Ther (2009) 8(3):665–71. doi: 10.1158/1535-7163.MCT-08-0823 PMC3041173 19240160

[58] Liu JQ Hu A Zhu J Yu J Talebian F Bai XF. CD200-CD200R pathway in the regulation of tumor immune microenvironment and immunotherapy. Adv Exp Med Biol (2020) 1223:155–65. doi: 10.1007/978-3-030-35582-1_8 PMC7339106 32030689

[59] Diskin B Adam S Soto GS Liria M Aykut B Sundberg B. BTLA(+)CD200(+) b cells dictate the divergent immune landscape and immunotherapeutic resistance in metastatic vs. primary pancreatic cancer. Oncogene. (2022) 41(38):4349–60. doi: 10.1038/s41388-022-02425-4 35948648

[60] Panowski SH Srinivasan S Tan N Tacheva-Grigorova SK Smith B Mak YSL. Preclinical development and evaluation of allogeneic CAR T cells targeting CD70 for the treatment of renal cell carcinoma. Cancer Res (2022) 82(14):2610–24. doi: 10.1158/0008-5472.CAN-21-2931 35294525

[61] Sugiura D Maruhashi T Okazaki IM Shimizu K Maeda TK Takemoto T. Restriction of PD-1 function by cis-PD-L1/CD80 interactions is required for optimal T cell responses. Sci (New York NY). (2019) 364(6440):558–66. doi: 10.1126/science.aav7062 31000591

[62] Bae J Accardi F Hideshima T Tai YT Prabhala R Shambley A. Targeting LAG3/GAL-3 to overcome immunosuppression and enhance anti-tumor immune responses in multiple myeloma. Leukemia. (2022) 36(1):138–54. doi: 10.1038/s41375-021-01301-6 PMC8727303 34290359

[63] Casazza A Laoui D Wenes M Rizzolio S Bassani N Mambretti M. Impeding macrophage entry into hypoxic tumor areas by Sema3A/Nrp1 signaling blockade inhibits angiogenesis and restores antitumor immunity. Cancer Cell (2013) 24(6):695–709. doi: 10.1016/j.ccr.2013.11.007 24332039

[64] González-Ochoa S Tellez-Bañuelos MC Méndez-Clemente AS Bravo-Cuellar A Hernández Flores G Palafox-Mariscal LA. Combination blockade of the IL6R/STAT-3 axis with TIGIT and its impact on the functional activity of NK cells against prostate cancer cells. J Immunol Res (2022) 2022:1810804. doi: 10.1155/2022/1810804 35465350 PMC9020142

